# Mapping cellular stress and lipid dysregulation in Alzheimer-related progressive neurodegeneration using label-free Raman microscopy

**DOI:** 10.1038/s42003-024-07182-6

**Published:** 2024-11-15

**Authors:** Annika Haessler, Michael Candlish, Jasmin K. Hefendehl, Nathalie Jung, Maike Windbergs

**Affiliations:** 1 Institute of Pharmaceutical Technology, Goethe University Frankfurt am Main, Frankfurt am Main, Germany; 2 Institute of Cell Biology and Neuroscience, Goethe University Frankfurt am Main and Buchmann Institute for Molecular Life Sciences, Frankfurt am Main, Germany

**Keywords:** Microscopy, Data processing, Neurodegeneration

## Abstract

Aβ plaques are a main feature of Alzheimer’s disease, and pathological alterations especially in their microenvironment have recently come into focus. However, a holistic imaging approach unveiling these changes and their biochemical nature is still lacking. In this context, we leverage confocal Raman microscopy as unbiased tool for non-destructive, label-free differentiation of progressive biomolecular changes in the Aβ plaque microenvironment in brain tissue of a murine model of cerebral amyloidosis. By developing a detailed approach, overcoming many challenges of chemical imaging, we identify spatially-resolved molecular signatures of disease-associated structures. Specifically, our study reveals nuclear condensation, indicating cellular degeneration, and increased levels of cytochrome c, showing mitochondrial dysfunction, in the vicinity of Aβ plaques. Further, we observe severe accumulation of especially unsaturated lipids. Thus, our study contributes to a comprehensive understanding of disease progression in the Aβ plaque microenvironment, underscoring the prospective of Raman imaging in neurodegenerative disorder research.

## Abstract (web summary)

Multivariate analysis of hyperspectral Raman imaging data unveils severe cellular toxicity and lipid dysregulation in the chemically complex Aβ plaque microenvironment.

## Introduction

Alzheimer’s disease (AD) is a severe progressive neurodegenerative disorder and with an aging population, AD has become a major global health challenge^1–3^. Despite tremendous efforts to understand AD, molecular mechanisms underlying the disease are not yet fully understood^4,5^. While accumulation of beta amyloid (Aβ) in extracellular plaques has long been, and still is, regarded as a pathological hallmark and primary cause of AD, recent studies have shifted their attention to other molecular alterations in AD, which are caused by Aβ and hence promote further deleterious downstream effects^6–11^. In this context, mitochondrial dysfunction is of increasing interest, since it is an early and well documented event in AD^12^. Numerous anomalies have been identified so far, such as a drastic decrease in cytochrome c oxidase (COX) function, increased occurrence of reactive oxygen species (ROS), and impaired energy metabolism^10,12,13^. Neuroinflammatory events, such as the activation of microglia, further lead to the accumulation of ROS and therefore contribute to cellular toxicity^14^. In addition, neuroinflammation and amyloid toxicity were also found to be mediated by the lipid metabolism, which is severely altered in brain tissue during AD^15,16^. Lipid peroxidation due to ROS has been reported already in early stages of AD, and recent approaches have been able to identify several genes associated with dysfunctional lipid metabolism, such as APOE4, which controls lipid homeostasis^17,18^. Although many aspects of these complex molecular mechanisms have been identified, comprehensive insight is still lacking. As AD comprises several intertwined processes on the tissue level, confocal imaging is a strategic approach to understand the pathophysiological tissue dynamics in a spatially-resolved manner. The most common technique used in this context is fluorescence microscopy. However, for visualization, the structures or compounds of interest need to be known and respective labels are required for differentiation. While many fluorescence labels for proteins as well as lipids are available, this approach only allows for the selective analysis of a limited number of known targets at the same time^19,20^. This is particularly disadvantageous if the molecular framework is still unknown. Moreover, the inherent complexity of these unexplored structures demands an imaging approach able to monitor molecular alterations without predetermined bias. In this context, an increasingly popular alternative is matrix-assisted laser desorption/ionization mass spectrometry imaging (MALDI-MSI). This label-free method can identify and resolve the location of multiple biomolecules at once^21,22^. However, the technique is often restricted by a very limited spatial resolution and considerable computational demands^23^. Thus, chemically selective techniques are urgently needed to understand and monitor pathophysiological events in tissue, whilst unveiling qualitative, as well as quantitative molecular information. In this context, confocal Raman microscopy has emerged as a promising approach to fill the gaps left by other technologies, as it is purely based on the inelastic scattering of light by the molecules in the sample^24^. The energy lost by the scattered light is distinct for each molecular bond present in a sample, thus resulting in a specific spectrum of scattered light (Raman spectrum), considered a molecular fingerprint^25,26^. In tissue samples, such molecular fingerprints contain signals derived from a multitude of biomolecules, allowing for simultaneous evaluation of various biomolecular characteristics without any labels, markers, or probes. Additionally, using a confocal setup enables the spatially-resolved acquisition of Raman spectra from pixels defined by their x-, y-, and z-position in the sample^27^. Thus, Raman microscopy has a high potential for the characterization of neurodegenerative disorders, both complimenting previous research and providing deeper insight into molecular changes^25,26,28,29^. However, applications of the technique to biological tissues are extremely difficult, due to issues such as autofluorescence, thermal damage and low signal-to-noise ratios of the acquired spectra^30^. Additionally, while tissue Raman peaks have been reported in literature, their identification in new sample matrices and relationship to disease associated structures is difficult to describe owing to the exceptionally complex ensuing data analysis^31,32^. In imaging data sets, challenges especially arise in the transitional regions between biological structures, blurring the border between them and thus impeding approaches such as PCA or cluster analysis. Furthermore, especially in the case of tissue Raman data, subtle shifts in the spectra may indicate grave alterations in chemical composition, complicating data analysis^30,32^. Hence, implementation of Raman microscopy in neurodegenerative research is still lacking, and so far, studies have been restricted to small sample sizes and limited statistical testing^33,34^. Furthermore, while Raman signatures of Aβ plaques and lipids have been reported, there is no detailed description of other spectral alterations or biological entities like cell nuclei or cytochrome c, although they reveal key information on neurodegenerative events in AD^33–40^.

Herein, we utilized Raman imaging to visualize cellular stress, mitochondrial dysfunction, and lipid dysregulation in the Aβ plaque microenvironment in the cerebral cortex, the starting point of amyloid pathology, during progressive neurodegeneration^7^. We conducted our study on hyperspectral data sets acquired from differently aged APP/PS1 mice (transgenic mice co-expressing KM670/671NL “swedish” mutated Amyloid precursor protein (APP) and L166P mutated Presenilin 1), an established model of AD, and wildtype (WT) mice^41^. After acquisition of the Raman data sets, we employed vertex component analysis (VCA), a multivariate algorithm for handling complex hyperspectral data sets, to process each scan^42^. This allowed for the calculation of biochemical abundance maps, providing spatial information for Aβ plaques, nuclei, cytochrome c, and lipids within the analyzed tissue and offering the foundation for further analysis of lipid distribution. To enable the statistical evaluation of the molecular information of Raman scans, we conducted principal component analysis, a method for visualizing spectral variance in fewer dimensions, and peak ratio analysis, a valuable tool for detecting subtle shifts in Raman spectra^43^. By focusing on the thorough development of a technical approach to such a large data set acquired from challenging sample matrices, this study aimed to establish a detailed, reproducible analytical method, advancing the application of confocal Raman microscopy to neurodegenerative research questions. Using this refined procedure, we also aimed to unravel comprehensive insight into the progression of AD in the Aβ plaque microenvironment by holistically characterizing and comparing Raman signatures of Aβ plaques, cell nuclei, cytochrome c and lipids for the first time.

## Results

**Fig. 1:**
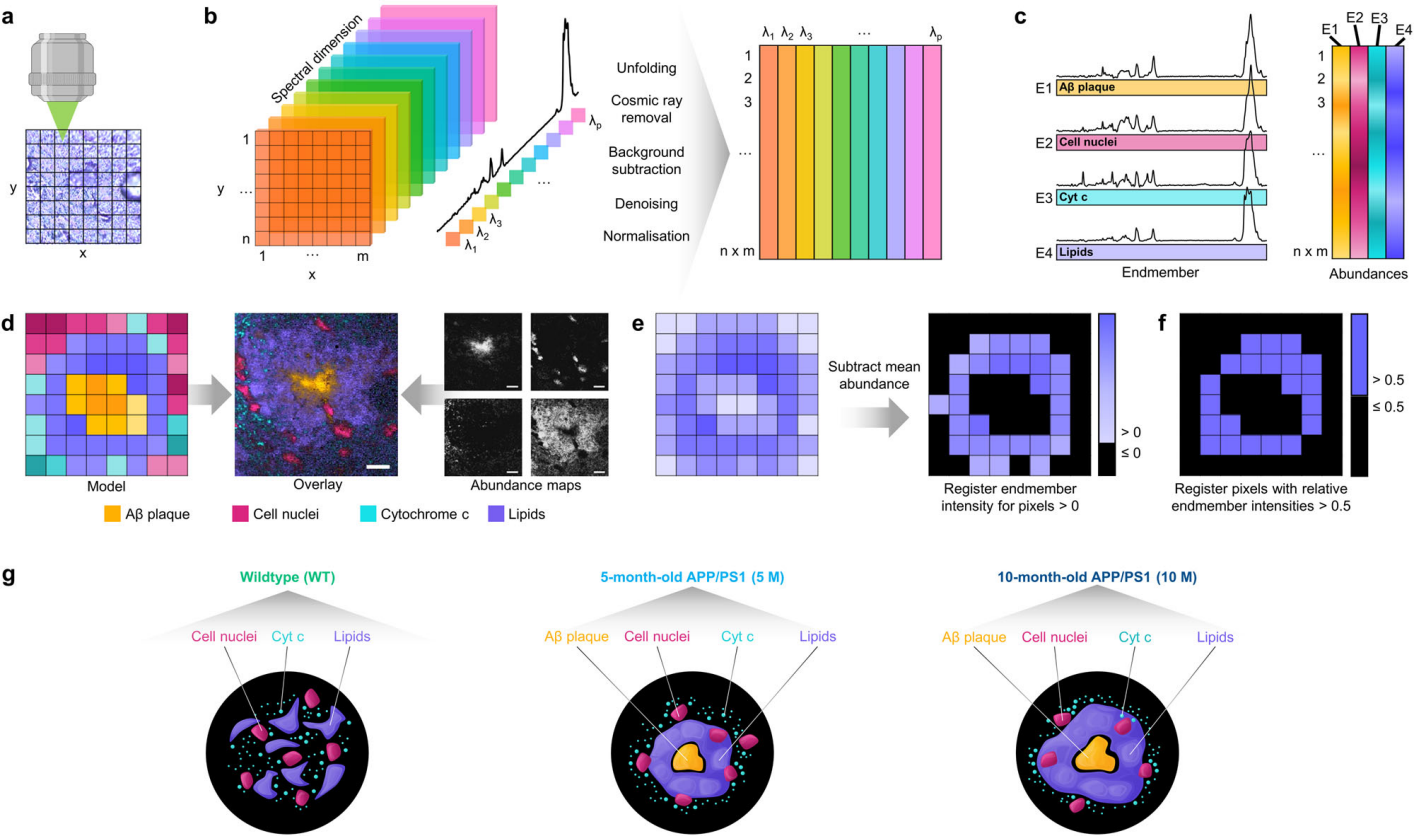
Methodological approach. **a** Raman imaging is performed by acquiring Raman spectra pixel by pixel. **b** The resulting 3D hyperspectral data set, defined by two spatial dimensions and one spectral dimension, is preprocessed, yielding an unfolded 2D matrix. **c** To elucidate chemical information, vertex component analysis is applied. Extreme Raman spectra in the data set, so-called endmembers, are assigned different biological entities. All remaining Raman spectra considered a linear mixture of these endmembers, allowing for the calculation of abundances of each endmember in the data set. **d** By mapping and overlaying these chemical abundances, distribution of biological entities in the scan can be visualized. **e** For distribution analysis, the mean abundance is subtracted from abundance maps of a chosen biological entity, e.g., lipids, pixels with an intensity >0 are counted, and their corresponding intensity values registered. **f** For the assessment of the area covered by a biological entity, pixels with relative abundances of that entity >0.5 are counted. **g** In the qualitative analysis, the Raman spectra of each biological entity extracted from scans of WT, 5 M and 10 M APP/PS1 mice are first analyzed using PCA and peak ratio analysis to evaluate reproducibility of the data analysis pipeline. For example, for 10 M APP/PS1 mice, highly correlated spectra of the Aβ plaque, cell nuclei, cytochrome c, and lipids were pooled to perform PCA and peak ratio analysis. In the next step, spectra are analyzed according to biological entity to closely examine differences between the healthy and diseased states in the tissue. For example, for lipids (purple), highly correlated lipid spectra from each disease stage (WT, 5 M APP/PS1 and 10 M APP/PS1) were pooled to perform PCA and peak ratio analysis.

With Raman analysis, we first acquired spatially-resolved molecular signatures of Aβ plaques, cell nuclei, cytochrome c, and lipids from the cortical gray matter of brain sections of differently aged APP/PS1 mice and a WT control group (Fig. 1a–c). Then, we closely investigated the biochemical nature and distribution of different biological entities in the Aβ plaque environment, assessing their alterations with respect to disease progression (Fig. 1d–g).

### Simultaneous detection of Aβ plaques, cell nuclei, cytochrome c, and lipids in tissue

**Fig. 2:**
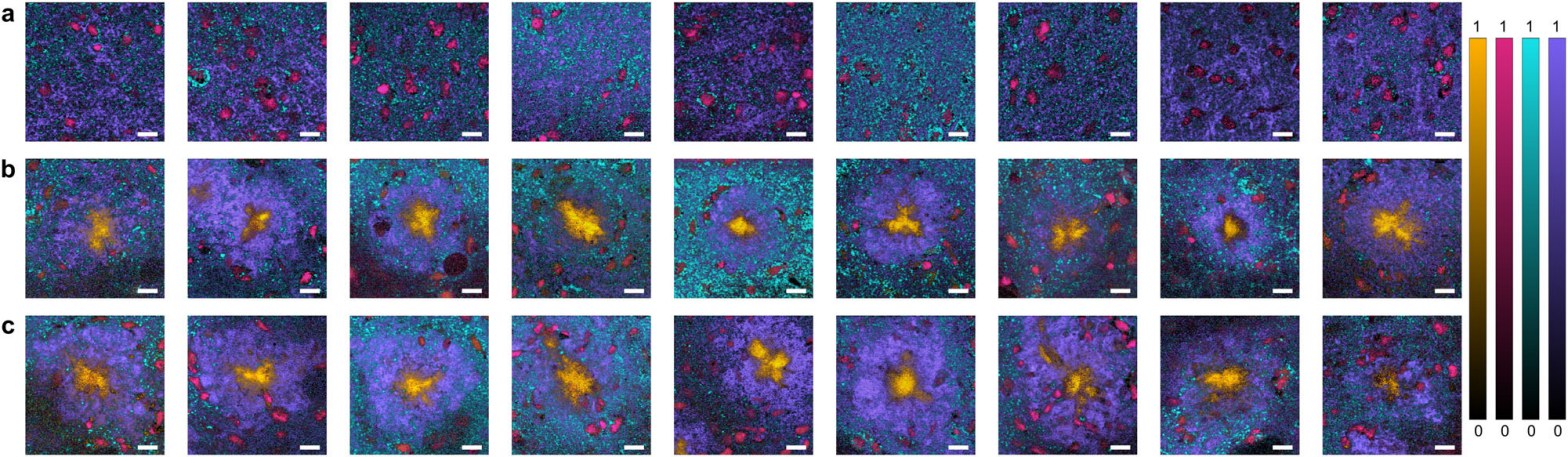
Semi-quantitative false color Raman images of healthy and diseased tissues. The rows present merged Raman images of (**a**) WT mice, (**b**) 5 M APP/PS1 mice, and (**c**) 10 M APP/PS1 mice showing Aβ plaques (yellow), cell nuclei (pink), cytochrome c (cyan), and lipids (purple). Scale bars depict 10 µm.

To achieve a label-free identification of distinct biological entities as a basis for our study, we first acquired hyperspectral data sets using Raman imaging. No changes in the raw Raman spectra implying thermal degradation over time were observed upon inspection. After preprocessing, pixels containing high levels of molecular structures indicative of Aβ plaques, cell nuclei, cytochrome c, or lipids were identified with vertex component analysis (VCA) in the data set. Since the Raman spectra of these pixels represent the purest possible Raman spectrum of the respective biological entity, they are also referred to as endmembers. Endmember signatures of Aβ plaques, cell nuclei, cytochrome c, and lipids were differentiated using their typical Raman peaks, such as the β-sheet peak at 1670 cm^−1^, DNA peaks at 785 cm^−1^ and 1341 cm^−1^, the heme Fe peak at 750 cm^−1^, and the intensity of -CH_2_ vibrations at 2850 cm^−1^, respectively. A detailed list of Raman peaks and their biological context is displayed in Supplementary Table 1^31,33^. After calculating the distribution of Aβ, cell nuclei, cytochrome c, and lipids in the tissue, false color Raman images were generated using an overlay of all investigated biological entities (Fig. 2). While the Raman images of the analyzed areas in the cortex of WT and 1.5 M APP/PS1 mice (Supplementary Fig. 1) did not contain any Aβ plaques, tissue of 5 M and 10 M APP/PS1 mice included this pathological AD hallmark. Furthermore, cell nuclei, cytochrome c, and lipids occurred evenly distributed in the tissue of WT mice. Conversely, images from 5 M and 10 M APP/PS1 mice showed a plaque-surrounding, thick lipid structure which appeared larger in the 10 M APP/PS1 mice. Cell nuclei and cytochrome c were located at the outer edges of the lipid structure in both these groups.

### Distribution of lipids in the Aβ plaque microenvironment

**Fig. 3:**
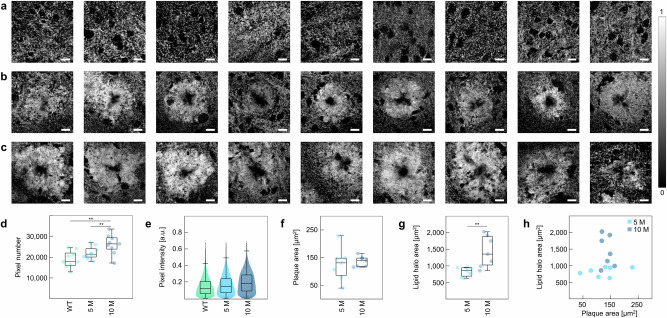
Analysis of lipid distribution and the spatial dimension of Aβ plaques and lipid halos. Lipid abundance maps of (**a**) WT mice, (**b**) 5 M APP/PS1 mice, and (**c**) 10 M APP/PS1 mice. **d** Number of pixels associated with lipids after background subtraction, *n* = 3, *N* = 3. **e** Lipid abundance intensities of all pixels left after background subtraction. **f** Measurement of the Aβ plaque area, *n* = 2–3, *N* = 3. **g** Measurement of the lipid halo area, *n* = 2–3, *N* = 3. **h** Lipid halo area plotted against plaque area. Statistical test in (**d**): One-way ANOVA, Tukey-Kramer post-hoc test. Statistical test in (**f**, **g**): student’s t-test. Statistical significance is indicated by *(p < 0.05), **(p < 0.01) or ***(p < 0.001). Box plot whiskers extend to the most extreme data points which are not considered outliers. Scale bars depict 10 µm.

Next, we assessed alterations of the distribution of lipids during AD progression with a special focus on the Aβ plaque microenvironment. For this, we first leveled the lipid abundance maps obtained via VCA by subtracting the mean lipid intensity of all included data sets. Then, the pixels with a remaining intensity greater than 0 were summed and their intensities registered. Fig. 3a–c shows the chemical abundance maps of lipids in the different samples, emphasizing a marbled texture in the WT mice and 1.5 M APP/PS1 mice (Supplementary Fig. 2) and the spatial clustering of lipids in 5 M and 10 M APP/PS1 mice. Overall, more pixels of the Raman images were associated with lipids in 5 M and 10 M APP/PS1 mice than in WT mice, indicating, like the abundance maps in Fig. 3a–c, an increased amount of lipids in the Aβ plaque vicinity (Fig. 3d). Moreover, the pixels from the 5 M and 10 M APP/PS1 mice also contained higher lipid intensities, as displayed by the upshifting of the swarm charts in Fig. 3e, suggesting an increased density of lipids as well. Next, to calculate the area of the Aβ plaques and lipid halos in 5 M and 10 M APP/PS1 mice, pixels mostly associated with Aβ plaques and lipids (abundance value greater than 0.5, Supplementary Fig. 3) were counted to calculate the corresponding covered scan area, respectively. While the Aβ plaque area did not increase between 5 M and 10 M mice (Fig. 3f), analysis of the lipid halo area showed a significant 1.74-fold expansion (Fig. 3g). Moreover, Aβ plaque area and lipid halo area were not correlated, as emphasized by the absence of a trend in Fig. 3h depicting paired observations of Aβ plaque and lipid halo areas.

### Molecular profiling of disease state

**Fig. 4:**
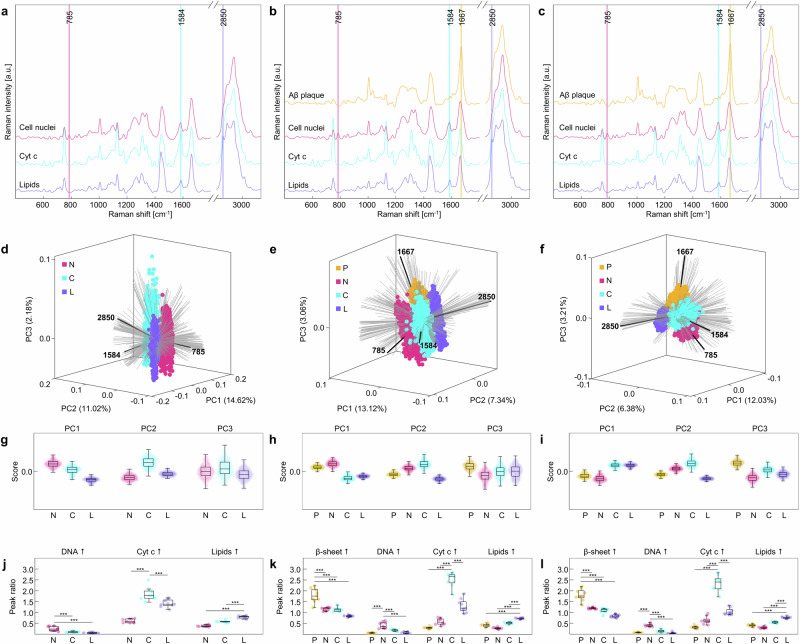
Qualitative analysis of Aβ plaques (yellow), cell nuclei (pink), cytochrome c (cyan), and lipids (purple) in WT, 5 M APP/PS1 mice, and 10 M APP/PS1 mice. Averaged Raman spectra of biological entities of (**a**) WT, (**b**) 5 M APP/PS1 mice, and (**c**) 10 M APP/PS1 mice, *n* = 3, *N* = 3. Markings highlight one characteristic peak of each entity (1667 cm^−1^ for Aβ plaques, 785 cm^−1^ for cell nuclei, 1584 cm^−1^ for cytochrome c (Cyt c), and 2850 cm^−1^ for lipids). PCA biplot for (**d**) WT, (**e**) 5 M APP/PS1, and (**f**) 10 M APP/PS1 mice with loadings indicating peaks typical for each biological entity in black. Corresponding PC scores are plotted as swarm charts for (**g**) WT, (**h**) 5 M APP/PS1, and (**i**) 10 M APP/PS1 mice, *n* = 3, *N* = 3. Peak ratio analysis of Raman spectra of biological entities for (**j**) WT, (**k**) 5 M APP/PS1, and (**l**) 10 M APP/PS1 mice using the ratios listed in Supplementary Table 2, *n* = 3, *N* = 3. Statistical test in (**j**, **k**): One-way ANOVA, Tukey-Kramer post-hoc test. Statistical significance is indicated by *(p < 0.05), **(p < 0.01) or ***(p < 0.001). Box plot whiskers extend to the most extreme data points which are not considered outliers. P = Aβ plaques, N = cell nuclei, C = cytochrome c, L = lipids.

To investigate the molecular nature of AD progression on tissue level, a qualitative analysis of spectral signatures was conducted in two steps. First, highly correlated Raman spectra of each endmember spectrum from each scan were extracted and mean spectra were calculated. In the second step, PCA and peak ratio analysis were performed on spectra arranged by disease state (WT, 5 M, 10 M). The peak ratios used in this part of the study are listed in Table S2, with a higher value for a given peak ratio indicating increased association with a biological entity. The results of the qualitative analysis are shown in Fig. 4. Since no Aβ plaques could be detected in WT mice tissue, only spectra assigned to the remaining biological entities, namely cell nuclei, cytochrome c, and lipids are plotted. All spectra varied visibly in several Raman shifts, but since the peak assignments for Raman spectra are extremely complex, spectra were only marked with one typical peak for each biological entity (Fig. 4a–c). A more detailed peak assignment is provided in Table S1.

Cell nuclei were identified via increased peaks at 785 cm^−1^ and 1338–1341 cm^−^^1^, 1371 cm^−1^, 1425 cm^−1^, and 1485 cm^−1^, related to DNA and RNA, but also increased protein abundances, at 2933 cm^−1^ (Supplementary Table 1, Fig. 4a–c). Cytochrome c spectra displayed specific peaks at 750 cm^−1^, 1129 cm^−1^, 1310 cm^−1^ and 1584 cm^−1^ (Supplementary Table 1, Fig. 4a–c). For lipids, peaks indicating cholesterol (702 cm^−1^), fatty acids (1070 cm^−1^), phosphate (1090 cm^−1^), esters (1740 cm^−1^), unsaturated lipids (1268 cm^−1^, 1650 cm^−1^, 3010–3015 cm^−1^) and saturated lipids (1444 cm^−1^, 2850 cm^−1^, 2883 cm^−1^) were identified (Supplementary Table 1, Fig. 4a–c)^31^. Aβ plaques exhibited increased peak intensities at 1005 cm^−1^, 1032 cm^−1^, and 1609 cm^−1^, 1238 cm^−1^, and 1667 cm^−1^, as well as 3060 cm^−1^, indicating the presence of phenylalanine, β-sheets, and amide bonds, respectively (Supplementary Table 1, Fig. 4b, c)^33^.

Differences in the spectra were further emphasized in the PCA score plot of each group. Using PCA, large data sets with numerous variables may be reduced to smaller sets consisting of fewer new variables, so called principal components (PCs). Therefore, influences of hundreds of Raman peaks can be visualized with only two or three PCs, allowing for the identification of data clusters. Furthermore, so-called loading vectors may be used to interpret PC plots. For example, positive values of the loading vector indicate a positive correlation with a PC, thereby conserving the original chemical information of the Raman spectra. In our analysis, within the 3D plot of each disease state, each biological entity formed a well separated cluster along the direction of a corresponding loading vector, representing peaks signifying Aβ plaques (if applicable), cell nuclei, cytochrome c and lipids at 1667 cm^−1^, 785 cm^−1^, 1584 cm^−1^, and 2850 cm^−1^, respectively (Fig. 4d–f, Supplementary Fig. 4). Further, as indicated by the PC swarm charts (Fig. 4g–i), WT and 5 M APP/PS1 mice varied especially in PC1 and PC2, whereas the 10 M APP/PS1 mice also varied strongly in PC3. Overall, the first three PCs accounted for 27.82% in WT, 23.52% in 5 M, and 21.62% of variance explained in 10 M APP/PS1 mice. To evaluate the discriminative power of Raman spectra between biological entities, we utilized peak ratio analysis of mean spectra of each scan (Fig. 4j–l). Specifically, we utilized the ratios listed in Table S2, which indicate increasing amounts of β-sheets, DNA, cytochrome c, and lipids, respectively^31,33^. For all disease states, the differentiation of biological entities is possible on a highly significant level. Similar results could also be acquired for 1.5 M APP/PS1 mice (Supplementary Fig. 5).

### Differentiation of disease states by biological entity

**Fig. 5:**
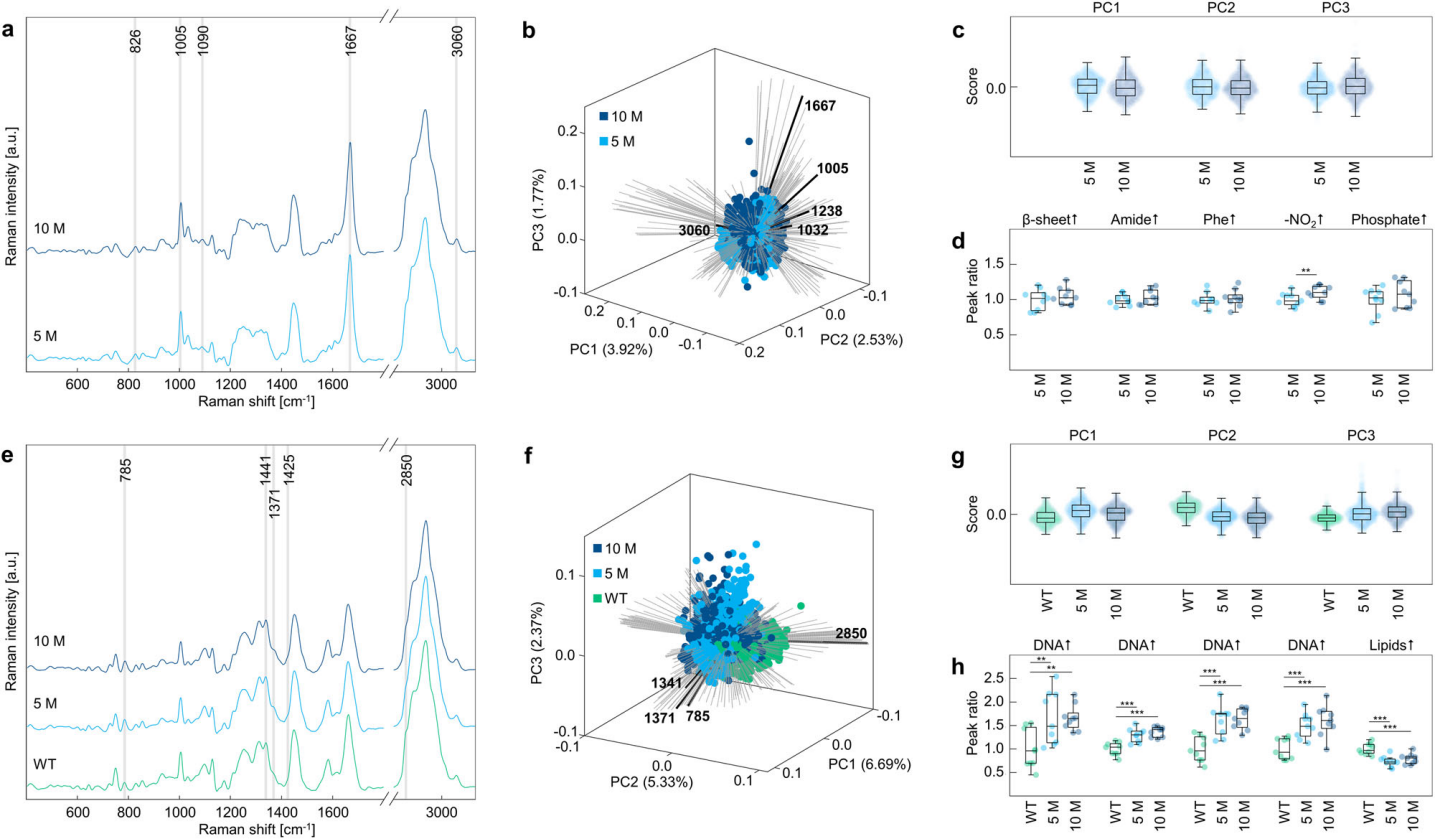
Qualitative analysis by biological entity. **a**–**d** Analysis of Raman spectra of Aβ plaques. **a** Raman spectra of Aβ plaques from 5 M (light blue) and 10 M APP/PS1 (dark blue) mice, *n* = 3, *N* = 3. Peaks of interest (Supplementary Table 1) are marked. **b** PCA biplot with loadings indicating peaks typical for Aβ plaque spectra in black and (**c**) PC scores plotted as swarm charts, *n* = 3, *N* = 3. **d** Peak ratios analysis of Raman spectra using ratios listed in Supplementary Table 3, n = 3, *N* = 3. **e**–**h** Analysis of Raman spectra of cell nuclei. **e** Raman spectra of cell nuclei from WT (green), 5 M (light blue), and 10 M APP/PS1 (dark blue) mice, *n* = 3, *N* = 3. Peaks of interest (Supplementary Table 1) are marked. **f** PCA biplot with loadings indicating peaks typical for cell nuclei spectra in black and (**g**) PC scores plotted as swarm charts, *n* = 3, *N* = 3. **h** Peak ratios analysis of Raman spectra using ratios listed in Supplementary Table 3. Statistical test in (**d**): Student’s t-test. Statistical test in (**h**): One-way ANOVA, Dunnett post-hoc test. Statistical significance is indicated by *(p < 0.05), **(p < 0.01) or ***(p < 0.001). Box plot whiskers extend to the most extreme data points which are not considered outliers.

**Fig. 6:**
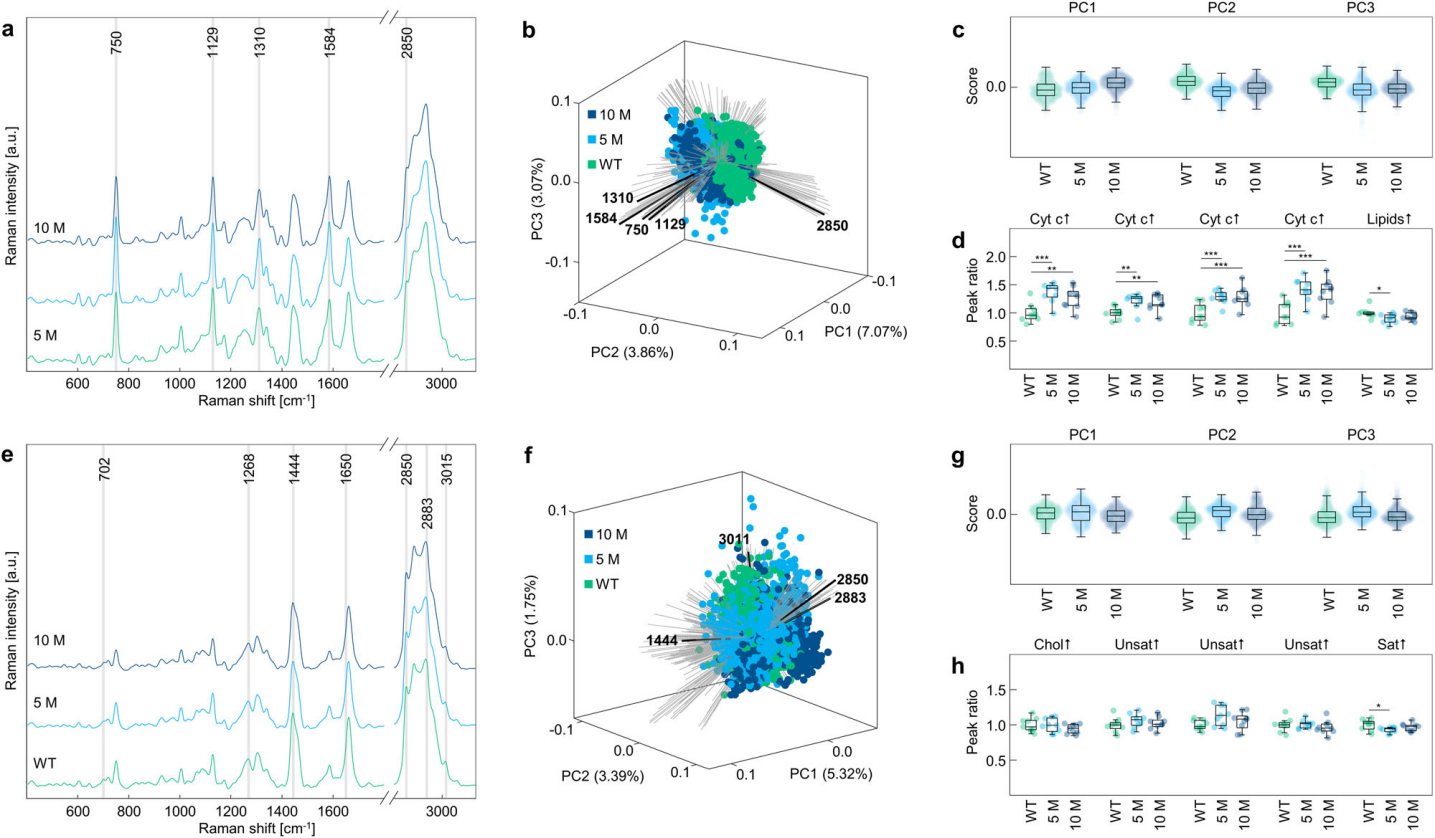
Qualitative analysis by biological entity. **a**–**d** Analysis of Raman spectra of cytochrome c. **a** Raman spectra of cytochrome c from WT (green), 5 M (light blue), and 10 M APP/PS1 (dark blue) mice, *n* = 3, *N* = 3. Peaks of interest (Supplementary Table 1) are marked. **b** PCA biplot with loadings indicating peaks typical for cytochrome c spectra in black and (**c**) PC scores plotted as swarm charts, *n* = 3, *N* = 3. **d** Peak ratios analysis of Raman spectra using ratios listed in Supplementary Table 3, n = 3, *N* = 3. **e**–**h** Analysis of Raman spectra of lipids, *n* = 3, *N* = 3. **e** Raman spectra of lipids from WT (green), 5 M (light blue), and 10 M APP/PS1 (dark blue) mice. Peaks of interest (Supplementary Table 1) are marked, *n* = 3, *N* = 3. **f** PCA biplot with loadings indicating peaks typical for lipid spectra in black and (**g**) PC scores plotted as swarm charts, *n* = 3, *N* = 3. **h** Peak ratios analysis of Raman spectra using ratios listed in Supplementary Table 3, *n* = 3, *N* = 3. Statistical test in (**d**, **h**): One-way ANOVA, Dunnett post-hoc test. Statistical significance is indicated by *(p < 0.05), **(p < 0.01) or ***(p < 0.001). Box plot whiskers extend to the most extreme data points which are not considered outliers. Cyt c cytochrome c, chol cholesterol, unsat unsaturation, sat saturation.

Through grouping the Raman spectra acquired from each disease state (WT, 5 M, 10 M) by biological entity, we aimed to monitor disease progression in the Aβ plaque microenvironment by investigating the spectral changes of different biological entities (Figs. 5 and 6; Supplementary Figs. 6 and 7). To achieve this, specific peak ratios signifying changes in structure, composition, or metabolic states, were chosen (Supplementary Table 3). Both spectra and PCA along loading vectors of typical Aβ peaks did not reveal any notable molecular changes in Aβ plaque spectra acquired from 5 M and 10 M APP/PS1 mice (Fig. 5a–c), and all remaining loadings did not vary strongly compared to loadings of the previous analysis (Supplementary Figs. S4 and 6). Moreover, only the peak ratio investigating nitration of Aβ at tyrosine residues (826 cm^−1^/2935 cm^−1^) significantly differed between 5 M and 10 M APP/PS1 mice (Fig. 5d). Spectra of cell nuclei (Fig. 5e) were also extremely similar throughout the disease states, however, PCA (Fig. 5f, g) revealed differences in PC1 (6.69%) and PC2 (5.33%) along loadings of DNA (785 cm^−1^, 1341 cm^−1^, 1371 cm^−1^) and lipids (2850 cm^−1^) between the disease states. Subsequent peak ratio analysis revealed a significant rise in DNA-related Raman peak intensities and a decrease of lipid peaks in cell nuclei Raman spectra of 5 M and 10 M APP/PS1 mice compared to WT mice (Fig. 5h). Similar trends concerning the differences in disease state were observable for the Raman signatures of cytochrome c (Fig. 6a). The spectra were extremely similar again, and differences were only revealed by PCA (Fig. 6b, c) in PC1 (6.50%) and PC2 (3.88%) along loadings of cytochrome c (750 cm^−1^, 1129 cm^−1^, 1310 cm^−1^ and 1584 cm^−1^) and lipids (2850 cm^−1^). Using peak ratio analysis, these results were affirmed by a rise of cytochrome c and a significant reduction in lipid peaks found in 5 M mice compared to WT mice (Fig. 6d). For 10 M APP/PS1 mice, the reduction of lipid peaks within the cytochrome c spectra was not significant. For lipids, differences were less clear, as both the spectra and the PCA did not reveal trends (Fig. 6e–g). However, with peak ratio analysis, subtle changes in lipid composition became apparent (Fig. 6h). Notably, we observed a slight decrease in cholesterol between 5 M and 10 M APP/PS1 mice, as well as a notable increase of relative unsaturation in lipids in the Aβ plaque microenvironment, however, both were found not be statistically significant. Peak ratio analysis using cell nuclei, cytochrome c, and lipid Raman spectra acquired from 1.5 M APP/PS1 mice showed no significant difference to WT mice (Supplementary Fig. 7).

## Discussion

Confocal Raman microscopy was employed to characterize molecular changes in the Aβ plaque microenvironment during Alzheimer’s disease (AD) progression in a mouse model of cerebral amyloidosis (Fig. 1). Importantly, the consistency of the results derived from our data, as well as the absence of indicators of heat degradation, for example due to resonance of cytochrome c molecular vibrations with the incident laser light, show that the imaging conditions were well-suited for the sample matrix^44,45^. Apart from these technical challenges, such uniform results could also only be obtained due to the confocal setup of the Raman microscope, allowing for spatially defining the pixels in which the Raman spectra were acquired. Confocal setups also minimize noise introduced by scattering effects of adjacent areas, which is especially important for vertex component analysis (VCA), since the algorithm searches for pixels in the data set representing distinct entities^27^. Moreover, owing to the contribution of a multitude of different molecules to a spectrum, the specificity of the technique may be potentially confounded. Therefore, the confocal setup with a resolution smaller than the biological structures to be imaged is essential to assure in the subsequent analysis that only highly correlated lipid spectra are used for the analysis of alterations. This way, we successfully obtained reproducible results in a large data set (Fig. 2, Supplementary Fig. 1) and achieved a clear differentiation between biological entities, including Aβ plaques, cell nuclei, cytochrome c, and lipids, in both healthy and diseased tissue (Fig. 4, Supplementary Figs. 4 and 5). These results were the basis for the subsequent analysis, in which we set out to explore the progression of AD and its implications for the manifestation of oxidative stress, mitochondrial dysfunction, and lipid dysregulation in the Aβ plaque microenvironment.

Our study confirms the signifying peaks proposed in literature of Raman spectroscopic analyses on Aβ plaques^33,40^. However, when comparing 5 M and 10 M APP/PS1 mice, we observed only minor changes in the spectrum and the size of the Aβ plaques (Figs. 3f and 5a–c). Specifically, we only detected a significant increase in nitration in the Aβ plaque Raman spectra, likely stemming from the inflammatory environment, although other modifications, such as phosphorylation, are reported for various murine models of AD as well (Fig. 5d)^46–50^. Possibly, the levels of other modified Aβ species are either too low or too large within plaques or the radiometric changes are simply too subtle. Further, plaque size substantially varied for 5 M APP/PS1 mice, and the lowest plaque areas were also measured in this cohort, aligning with previous literature reporting only moderate plaque growth and considerable variance in plaque size^51^. Consequently, confocal Raman microscopy possesses limitations in characterizing Aβ plaques, whilst remaining useful and reliable in their identification.

The recent paradigm shift in the research landscape has directed the focus away from the Aβ plaque itself, and toward the toxic microenvironment containing Aβ oligomers^52,53^. Several pathophysiological processes, such as oxidative stress, mitochondrial dysfunction, and lipid dysregulation unravel in this toxic Aβ plaque vicinity and are at the center of current research. Therefore, we analyzed the microenvironment of Aβ plaques to assess the potential of confocal Raman microscopy to detect AD-associated cellular stress, which is known to accompany and even precede plaque formation in AD^54–56^. To examine these alterations, we investigated the spectral signatures of cell nuclei and cytochrome c and were able to describe AD-related alterations in these Raman signatures (Figs. 5 and 6, Supplementary Fig. 6). Specifically, we observed elevated DNA levels indicating nuclear condensation, as well as decreased lipid peaks in cell nuclei in 5 M and 10 M APP/PS1 tissue, suggesting cellular dysfunction and death (Fig. 5e–h)^57–59^. While cellular degeneration has been observed in previous studies using different methods, confocal Raman microscopy has so far primarily been used in rodent and human tissue to characterize Aβ plaques, proteins, and lipids, but not cell nuclei and cytochrome c^33,35–38,60^. Thus, this study describes a potential target for spectroscopic studies to investigate pathological alterations in AD. Remarkably, Raman peak ratios signifying cellular dysfunction remain unchanged between 5 M and 10 M APP/PS1 mice, indicating that toxic events must be initiated in earlier stages of the disease (Fig. 5h). Moreover, peak ratios calculated from Raman spectra acquired from 1.5 M APP/PS1 mice showed strong parallels to WT mice, confirming the disease-associated spectral signatures observed in 5 M and 10 M mice (Supplementary Fig. 7). Furthermore, VCA revealed cytochrome c signals located in small, ellipsoid structures in the scanned areas, indicating compartmentalization in mitochondria (Fig. 2). Additionally, significant enhancement of peak ratios indicative of cytochrome c and a decrease of lipid signals in APP/PS1 mice aged 5 months and 10 months was detected (Fig. 6a–d). Rises in cytochrome c are generally considered amplifiers of apoptosis, matching the results obtained from the analysis of cell nuclei Raman spectra^61^. Furthermore, these findings are also supported by previous studies reporting reduced activity of COX, the enzyme catalyzing the electron transfer from cytochrome c bound iron to oxygen in the last step of the respiratory chain, in AD^62,63^. Furthermore, mitochondrial dysfunction results in increasing levels of ROS, explaining the reduction of lipid peak ratios which imply lipid peroxidation due to oxidation of saturated acyl chains^10^. Furthermore, Raman peaks of cytochrome c have been used to describe other conditions, such as brain and breast cancers, suggesting cytochrome c Raman signals as general indicators of cellular dysfunction in disease^44,64,65^. The high sensitivity of Raman spectroscopic techniques for the detection of changes in DNA packing and cellular stress in AD tissue highlight the potential of Raman microscopy as a tool for monitoring progressive tissue damage in a neurodegenerative context. Although the transferability of the approach to other neurodegenerative diseases has not been proven so far, the presence of common principles (inflammatory environment, mitochondrial dysfunction), e.g., in glioma and Parkinson’s disease, shows that the implementation of Raman imaging in other pathological contexts may also provide insight into progressive alterations^66,67^.

In recent years, dysregulations in lipid metabolism have been indicated as key players in AD^16,29^. So far, lipid aggregations around plaques, known as lipid halos, have only been detected with chemical imaging, however, an in-depth analysis addressing distribution and biochemical composition of plaque-associated lipids during AD progression has not been accomplished^33^. Previously, cholesterol, sphingomyelin, and unsaturated fatty acids were identified in the vicinity of plaques in AD mouse models, whilst in humans, minor contributions of cholesterol, but considerable levels of unsaturated lipids were found, highlighting the need for additional research^33,40,60^. In our study, we first revealed an increasing abundance and density of lipids in the tissue, as well as the formation and growth of lipid halos in the vicinity of Aβ plaques (Fig. 3, Supplementary Fig. 3). While plaque size remained relatively stable between 5 M and 10 M APP/PS1 mice, we observed a significant increase in lipid halo size, indicating local toxicity most likely caused by the accumulation of soluble Aβ oligomers in the parenchyma around the plaque^68,69^. However, Aβ oligomers were not detected with the implemented approach, and no spatial alterations of lipids were present in 1.5 M APP/PS1 mice, again indicating absence of disease manifestation (Supplementary Fig. 2). While lipid halos have been detected in mouse brain tissue before, their characterization has been limited to univariate approaches or have lacked depth as compared to the lipid distribution analysis presented here^33,35^. Furthermore, studies using human tissue often do not detect lipid halos^36–38^. However, these studies have not identified cell nuclei as well, although they are often found in the vicinity of Aβ plaques^70^. Therefore, lipid halos and other biological entities in human tissue may go undetected due to the higher autofluorescence compared to rodent tissue^71^. Using another label-free approach, namely coherent anti-stokes Raman scattering, lipid aggregates consisting of long acyl chains and only little cholesterol have been found associated with Aβ plaques in human tissue^60^. Thus, the detection and characterization of lipid halos in human tissue, as well as their comparison to lipid halos found in mouse brain tissue, remain a challenge and requires further research. Concerning qualitative analysis of lipids, unsaturation increased between WT and 5 M APP/PS1 mice, while slight decreases of cholesterol and unsaturated lipids were detected when comparing 5 M and 10 M APP/PS1 mice (Fig. 6e–h). Although these are not statistically significant trends, except the ratio of 2850 cm^−1^/2935 cm^−1^, they were extremely consistent, despite having been calculated from thousands of Raman spectra acquired from different animals. So far, decreases of cholesterol in whole brain homogenates and isolated lipid rafts of differently aged APP/PS1 mice have been detected, aligning with our results when comparing 5 M and 10 M APP/PS1 mice^72^. However, especially cholesterol is regarded as a main feature of AD and has been found to potentiate Aβ toxicity, making decreases in cholesterol seem contradictory^73^. Yet, other research suggests that changes in cholesterol levels are not only specific for brain regions, but that alterations of cholesterol content may be a cause, but also a consequence of Aβ toxicity^74^. Similarly, the biological context of unsaturated lipid levels is still unclear due to their ambiguous role in AD. Whilst many publications link them to anti-inflammatory phenotypes of microglia, a closer look reveals a more complex interplay^75,76^. Evidently, both unsaturated and saturated lipids experience an increase in abundance in brain tissue of AD patients.^8^ Furthermore, unsaturated lipids, depending on structure and degree of unsaturation, may also be strongly pro-inflammatory, such as arachidonic acid and its metabolites^8,15,17^. Taking the pathological process in the tissue into account, the increased amount of unsaturated lipids found in the Aβ plaque microenvironment 5 M APP/PS1 mice compared to WT mice indicates a strong inflammatory response, matching our previous observations concerning cellular stress and mitochondrial dysfunction. Taken together, Aβ plaques were detectable in the tissue of both 5 M and 10 M APP/PS1 mice, with both exhibiting approximately equally strong indications of cellular stress and degeneration according to peak ratio analysis. However, while lipid halos were significantly larger in 10 M than in 5 M APP/PS1 mice, plaque size stagnated, suggesting local toxicity exerted by soluble amyloid oligomers surrounding Aβ plaques. Moreover, since we could not find any of such alterations in 1.5 M APP/PS1 mice (Supplementary Figs. 1, 2, 5, 7), disease exacerbation in the Aβ plaque microenvironment must occur in early stages of plaque development and deposition. Therefore, an important challenge for future studies using Raman imaging to investigate earlier stages of Aβ plaque development will be identifying immature Aβ plaques. According to our results, such areas cannot be identified via white light, and thus require additional imaging approaches and strategies.

So far, the difficulty of Raman data acquisition and analysis has hindered the implementation of confocal Raman microscopy and confined possible synergies with other imaging approaches in AD research. However, by comparing multiple biological structures during disease progression in the Aβ plaque microenvironment, this study has paved the way for investigating various new research questions. These research fields include the application of our approach to other brain regions affected by AD or the analysis of comorbidities of AD, like vascular dysfunction^77^. Such investigations may add depth to our current knowledge by simultaneously spatially resolving changes in targets which are difficult to stain, such as cellular stress and lipids, potentially providing entirely new chemical information. These future studies will be facilitated by the exemplary, highly reproducible technical approach presented here. Moreover, due to the unique label-free, non-destructive approach, confocal Raman microscopy can be combined with other imaging approaches to achieve complimentary insights. Specifically, confocal Raman imaging may synergize with subsequent fluorescence microscopy, allowing for co-localized analysis of the sample with two techniques, which is useful especially in explorative settings and potentially identifying disease targets. Further synergies of our approach are also possible in spatially resolving smaller molecular architectures which cannot be imaged with other label-free techniques, such as MALDI-MSI for example due to restrictions in resolution^23^.

The approach presented here demonstrates the advantages of confocal Raman microscopy as high-resolution chemical imaging technique for the thorough spatial analysis of pathological tissue alterations. Therefore, this study also paves the way for synergistic unions with complementary imaging techniques like fluorescence microscopy to elucidate neurodegenerative diseases, their etiology, and potential treatment. Hence, this work presents a valuable advancement not only in the implementation of confocal Raman microscopy into complex sample matrices, but also in the quest for a profound understanding of AD disease pathology and neurodegenerative disorders in general.

## Methods

### Animal models and sample preparation

The APP/PS1 mouse model of cerebral amyloidosis (transgenic mice co-expressing KM670/671NL “swedish” mutated Amyloid precursor protein (APP) and L166P mutated Presenilin 1) was used in this study^41^. Animal experiments and husbandry were approved by the animal welfare committee of the Johann Wolfgang Goethe University (ethical committee) and the Regierungspräsidium Darmstadt (board) (protocol numbers FR1001/FR2018/FR2010). Four animal groups were defined: healthy control mice aged 5 months (wildtype, WT), APP/PS1 mice aged 1.5 months (1.5 M APP/PS1), aged 5 months (5 M APP/PS1), and aged 10 months (10 M APP/PS1), respectively. Mice were sacrificed with an overdose of isoflurane, transcardially perfused with 20 ml of room temperature phosphate-buffered saline (PBS) followed by 20 ml of ice-cold 4% phosphate-buffered paraformaldehyde (PFA, pH 7.4). Brains were subsequently dissected and post-fixed for 2 h in 4% PFA on ice then left overnight (or until the brains had sunk) in 30% sucrose in PBS at 4 °C. Brains were then frozen in liquid nitrogen and 40 µm free-floating sections prepared using a sliding microtome (Slee medical GmbH, Nieder-Olm, Germany). Prepared sections were stored free-floating in freezing solution (10x PBS, ethylene glycol, glycerol and water in a volume ratio of 1:3:3:3) at −20 °C. On the day of analysis, the respective section was washed for 15 min in PBS under gentle shaking before being placed on a calcium fluoride glass dish. After a wash with MilliQ water, the section was left to dry. Raman imaging was performed within 24 h after the section was prepared.

### Raman imaging

Raman imaging in the cerebral gray matter of the brain sections was conducted with a confocal Raman microscope WITec Alpha300R+ (WITec GmbH, Ulm, Germany) equipped with a 532 nm laser set to the power of 5 mW in front of the 50x objective (NA 0.8). Scans were acquired with 0.3 s integration time in areas sized 70 × 70 µm with a resolution of 0.333 µm (44,100 spectra per scan). Laser power and integration time were optimized to avoid burning of the tissue. Similar parameters were also employed by other groups, and thermal damage was only observed by longer exposure times (>20–60 s at 5 mW), or higher laser powers (10–30 mW). Nevertheless, each scan was checked for thermal damage artifacts, observable by changes in peak position and width, after imaging in the WITec Project FOUR software (WITec GmbH, Ulm, Germany) to ensure data quality^78^. The settings above enabled bleaching of the autofluorescence of each line of consecutive pixels by the line of pixels before it. To also enable this for the first line of pixels, we performed a line scan adjacent to this first line of pixels of the imaging area. For 5 M and 10 M APP/PS1 mice areas including an Aβ plaque and its surrounding tissue were chosen for imaging. The areas were chosen via white light images and confirmed with a Raman spectrum taken in the center of a suspected Aβ plaque. For 1.5 M APP/PS1 and WT mice, randomly chosen areas in the cortical gray matter were imaged since the tissue did not contain any Aβ plaques identifiable via white light imaging. Three Raman scans were recorded from 1 to 2 sections from each mouse, resulting in 36 individual Raman scans (1,587,000 spectra in total, 9 per group, *n* = 3, *N* = 3).

### Data preprocessing

Background subtraction (“shape” function using a rolling circle background subtraction, window size set to 100) and cosmic ray removal were performed in the Project Four software (WITec GmbH, Ulm, Germany). Next, scans were exported to Matlab (The MathWorks Inc., Natick, USA), where all further preprocessing steps were conducted. Briefly, each scan was smoothed using an Savitzky-Golay filter (order: 3, window size: 9) and normalized using the Standard-Normal-Variate method.

### Vertex component analysis

As a linear spectral unmixing algorithm, vertex component analysis (VCA) assumes the presence of pure pixels, also called endmembers, in a hyperspectral data set, whilst all remaining pixels are considered linear mixtures of these endmembers. In a biological setting, Raman endmember spectra represent different biological entities, such as Aβ plaque, cell nuclei, cytochrome c or lipids, whose spectral signatures are purest at a specific location (Fig. 1c). Using these signatures, abundance maps for each endmember spectrum can be derived from the hyperspectral data (Fig. 1d). Abundances in this context are simply varying pixel intensities of each biological entity. The data sets investigated here were processed separately with VCA using the implementation by Schmidt et al. in Matlab (Version 2022a, The MathWorks Inc., Natick, USA)^79^. First, the number of endmembers in each data set was estimated using the noise-whitened Harsanyi–Farrand–Chang (NWHFC) method. Then, VCA was performed to find the endmember signatures. Using these, individual abundance maps for Aβ plaques, cell nuclei, cytochrome c, and lipids as well as an overlay were calculated.

### Analysis of distribution of lipids in tissue

The analysis was conducted based on the endmember abundance maps of the lipid endmember (Fig. 1e). First, the mean abundance of lipids of all data sets was subtracted from the lipid abundance map of each scan to level the data. This way, pixels which are not associated with lipids above a background level receive negative intensity values. Then, a matrix containing the lipid endmember intensity for remaining intensities >0 and 0 for pixels with intensities <0 was generated for each scan. Using this matrix, the number of pixels associated with lipids, which are left after data leveling, and a distribution of their intensities was acquired.

### Analysis of plaque and lipid halo size

Similar to the analysis above, two matrices were generated for each scan to collect pixels dominated by the Aβ plaque or lipid endmember respectively (>50% correlation, Fig. 1f). In contrast to the analysis before, intensities were not registered. Instead, a binary matrix with values >1 was generated. By summing all pixels with a value of 1 in the binary matrix and multiplying the sum with the pixel area of 0.110889 µm^2^, the area of Aβ plaques and lipid halos was calculated. The analysis was only conducted for the quantification of Aβ plaque and lipid halo area for 5 M and 10 M APP/PS1, as the other groups do not exhibit these features. In both investigated groups, data sets were excluded from the analysis if no coherent lipid halo was visible or multiple plaques were covered in one scan.

### Principal component analysis

For each scan, an equal number of highly correlated spectra from each biological entity needed to be extracted to conduct a comparable PCA and qualitative analysis. Considering the differing amount of noise in the data sets, using a certain percentage cutoff value (for example >95% correlation with the endmember) was not a viable option, as this resulted in only few spectra extracted for noisier data sets. Thus, a fixed number of correlated spectra were extracted instead. In this case, we chose a cutoff value of 100 spectra, since this cutoff is small enough to still extract highly correlated spectra in endmembers with only low abundance and delivers enough spectra to obtain interpretable mean spectra. After extracting the 100 most correlated spectra of each biological entity (Aβ plaques, cell nuclei, cytochrome c, and lipids), principal component analysis (PCA) was conducted in two arrangements (Fig. 1g): In the first arrangement, spectra were grouped according to biological entity per disease state (1.5 M, 5 M, and 10 M APP/PS1) and control (WT), and the PCA applied separately to each group. In the second arrangement, spectra were grouped according to disease state per biological entity with again a separately applied PCA. This way, the PCAs in the first arrangement analyze the differences between biological entities inside each disease state, whilst the second arrangement analyzes the differences between the disease states with respect to biological entity.

### Peak ratio analysis

For peak ratio analysis, we first calculated for each scan the mean spectrum of each biological entity using the 100 most correlated spectra, respectively. This yielded four different spectra for each scan of 5 M and 10 M APP/PS1 mice (Aβ plaque, cell nuclei, cytochrome c, and lipids) and three different spectra for each scan of 1.5 M APP/PS1 and 5 M WT mice (cell nuclei, cytochrome c, and lipids). Subsequently, peak ratio analysis was conducted on these mean spectra. For each peak ratio, we used a first peak which was correlated with a specific biological structure, and divided this peak by a second peak which was constant or inversely correlated for the respective biological entity. Peak ratios comparing biological entities between different disease states were normalized to the WT control to achieve a relative comparison of diseased Aβ plaque microenvironment to healthy tissue.

### Statistics and reproducibility

To test for statistical significance in the distribution analysis of lipids, one-way ANOVA followed by Tukey-Kramer post-hoc test or student’s two-tailed t-test was applied (indicated in caption of Fig. 3). For peak ratio analysis of spectra grouped by disease state, one-way ANOVA, followed by Tukey-Kramer post hoc-test was applied (indicated in caption of Fig. 4 and Supplementary Fig. S4). For peak ratio analysis of spectra grouped by biological entity, one-way ANOVA, followed by Dunnett post hoc test, or student’s two-tailed t-test were applied (indicated in caption of Fig. 6). Statistical significance is indicated by *(p < 0.05), **(p < 0.01), or ***(p < 0.001).

### Reporting summary

Further information on research design is available in the Nature Portfolio Reporting Summary linked to this article.

## Supplementary information

Supplementary Information

Description of Additional Supplementary Files

Supplementary Data

Reporting Summary

## Data Availability

All data needed to evaluate the conclusions in the paper are present in the paper and/or the Supplementary Information. Data used to plot the graphs in the main text and Supplementary Information are included in Supplementary Data. Raman data are available in the public repository Open Science Framework under the identifier https://osf.io/9fnru.
