# Supplementary material for: Mapping cellular stress and lipid dysregulation in Alzheimer-related progressive neurodegeneration using label-free Raman microscopy
Source: Commun Biol. 2024 Nov 15;7:1514. doi: 10.1038/s42003-024-07182-6 (PMC11568221; doi:10.1038/s42003-024-07182-6)
Supplement: Supplementary file 1 — Supplementary Information [file 42003_2024_7182_MOESM1_ESM.pdf]

Supplementary Information

Mapping cellular stress and lipid dysregulation in Alzheimer-related progressive neurodegeneration using label-free Raman microscopy

Authors
Annika Haessler[a], Michael Candlish[b], Jasmin K Hefendehl[b], Nathalie Jung[a], Maike Windbergs[a,*]

Affiliation
[a]Institute of Pharmaceutical Technology, Goethe University Frankfurt am Main, 60438 Frankfurt am Main, Germany

[b]Institute of Cell Biology and Neuroscience, Goethe University Frankfurt am Main and Buchmann Institute for Molecular Life Sciences, 60438 Frankfurt am Main, Germany

*Corresponding author, windbergs@em.uni-frankfurt.de

List of supplementary material

Supplementary Figure 1.	Semi-quantitative false color Raman images of imaged areas in diseased tissue of 1.5 M APP/PS1 mice.
Supplementary Figure 2.	Lipid abundance maps of imaged areas of 1.5 M APP/PS1 mice.
Supplementary Figure 3.	Binary images showing pixels counted for the analysis of the area covered by Aβ plaques and lipid halos.
Supplementary Figure 4.	Loadings of the PCA of Raman spectra grouped by disease state.
Supplementary Figure 5.	Raman analysis of Raman spectra acquired from 1.5 M APP/PS1 mice.
Supplementary Figure 6.	Loadings of PCA of Raman spectra grouped by biological entity.
Supplementary Figure 7.	Peak ratio analysis for Raman spectra acquired from 1.5 M APP/PS1 mice.
Supplementary Table 1.	Detailed list of Raman peak assignments.
Supplementary Table 2.	Peak ratios for the analysis of Raman spectra by disease state (WT, 1.5 M, 5 M and 10 M APP/PS1).
Supplementary Table 3.	Peak ratios for the analysis of Raman spectra by biological entity.
Supplementary references.	

Supplementary Figure 1

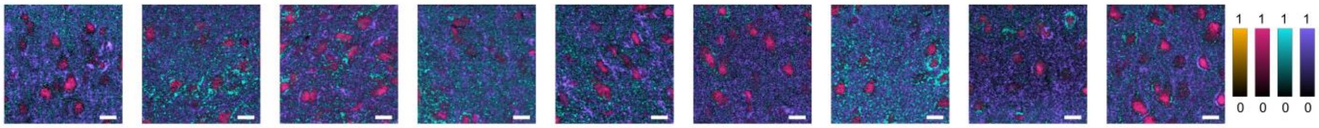

Supplementary Figure 1. Semi-quantitative false color Raman images of imaged areas in diseased tissue of 1.5 M APP/PS1 mice. The row shows a total of nine scans, acquired from three mice (three scans each) and depict the distribution of cell nuclei (pink), cytochrome c (cyan), and lipids (purple). Scale bars depict 10 µm.

Supplementary Figure 2

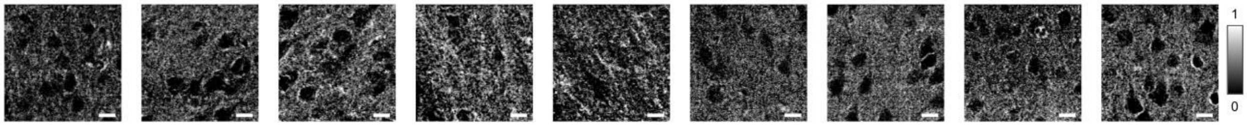

Supplementary Figure 2. Lipid abundance maps of imaged areas of 1.5 M APP/PS1 mice. The row shows a total of nine scans, acquired from three mice (three scans each). Scale bars depict 10 µm.

Supplementary Figure 3

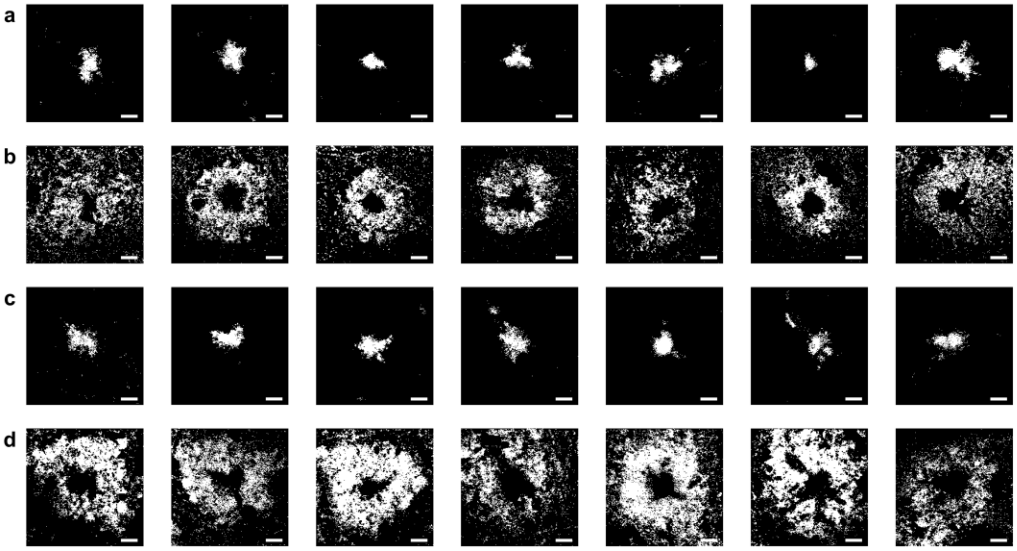

Supplementary Figure 3. Binary images showing pixels counted for the analysis of the area covered by Aβ plaques and lipid halos. (a) Aβ plaques of 5 M APP/PS1 mice. **(b)** Lipid halos of 5 M APP/PS1 mice. **(c)** Aβ plaques of 10 M APP/PS1 mice. **(d)** Lipid halos of 10 M APP/PS1 mice. Scale bars depict 10 µm.

Supplementary Figure 4

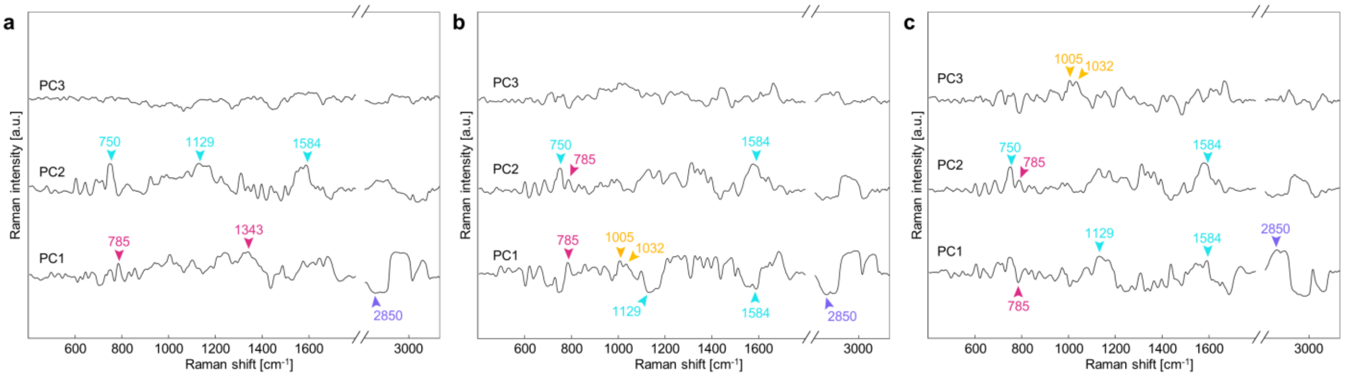

Supplementary Figure 4. Loadings of the PCA of Raman spectra grouped by disease state. Spectra of **(a)** WT, **(b)** 5 M APP/PS1, and **(c)** 10 M APP/PS1 mice are shown, *n* = 3, *N* = 3. Notable Raman peaks indicating a biological entity are marked with yellow (Aβ plaque), pink (cell nuclei), cyan (cytochrome c), and purple (lipids) arrow tips.

Supplementary Figure 5

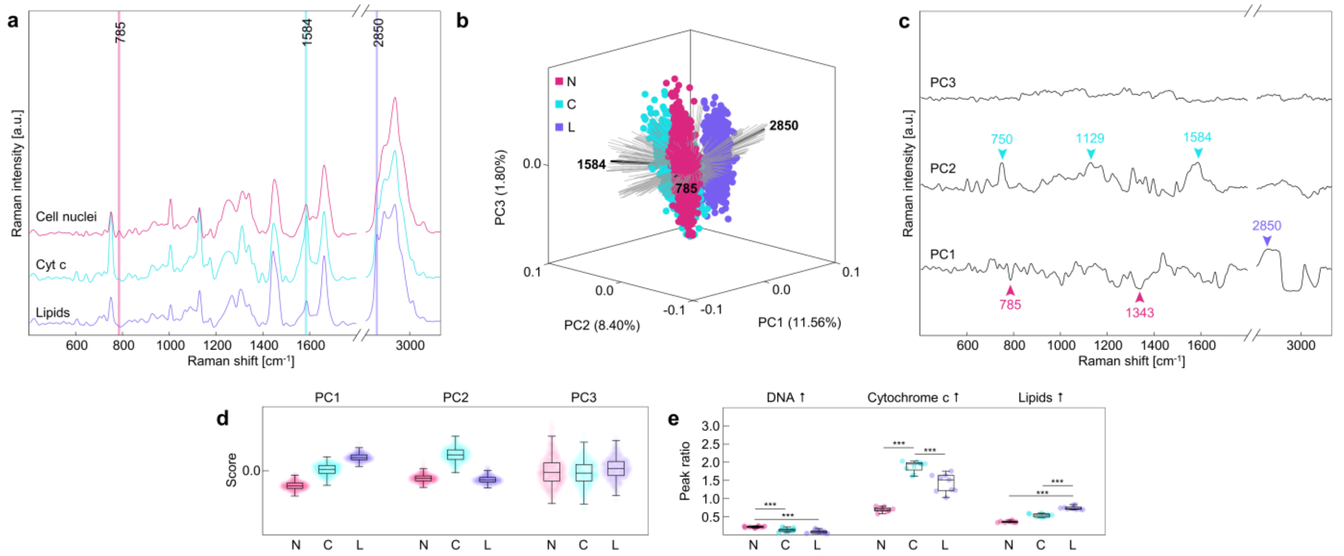

Supplementary Figure 5. Raman analysis of Raman spectra acquired from 1.5 M APP/PS1 mice. (a) Raman spectra of cell nuclei (pink), cytochrome c (cyan) and lipids (purple), *n* = 3, *N* = 3. Markings highlight one characteristic peak of each entity (785 cm^{-1} for cell nuclei, 1584 cm^{-1} for cytochrome c and 2850 cm^{-1} for lipids). **(b)** PCA biplot with loadings indicating peaks typical for each biological entity in black, *n* = 3, *N* = 3. **(c)** Loadings of the PCA. Notable Raman peaks indicating a biological entity are marked with pink (cell nuclei), cyan (cytochrome c), and purple (lipids) arrow tips, *n* = 3, *N* = 3. **(d)** PC scores plotted as swarm charts, *n* = 3, *N* = 3. **(e)** Peak ratio analysis of Raman spectra of biological entities using the ratios listed in Supplementary Table 2, *n* = 3, *N* = 3. Statistical test in J-K: One-way ANOVA, Tukey-Kramer post-hoc test. Statistical significance is indicated by *($p < 0.05$), **($p < 0.01$) or *** ($p < 0.001$). Box plot whiskers extend to the most extreme data points which are not considered outliers. cyt c = cytochrome c, N = Cell nuclei, C = Cytochrome c, L = Lipids.

Supplementary Figure 6

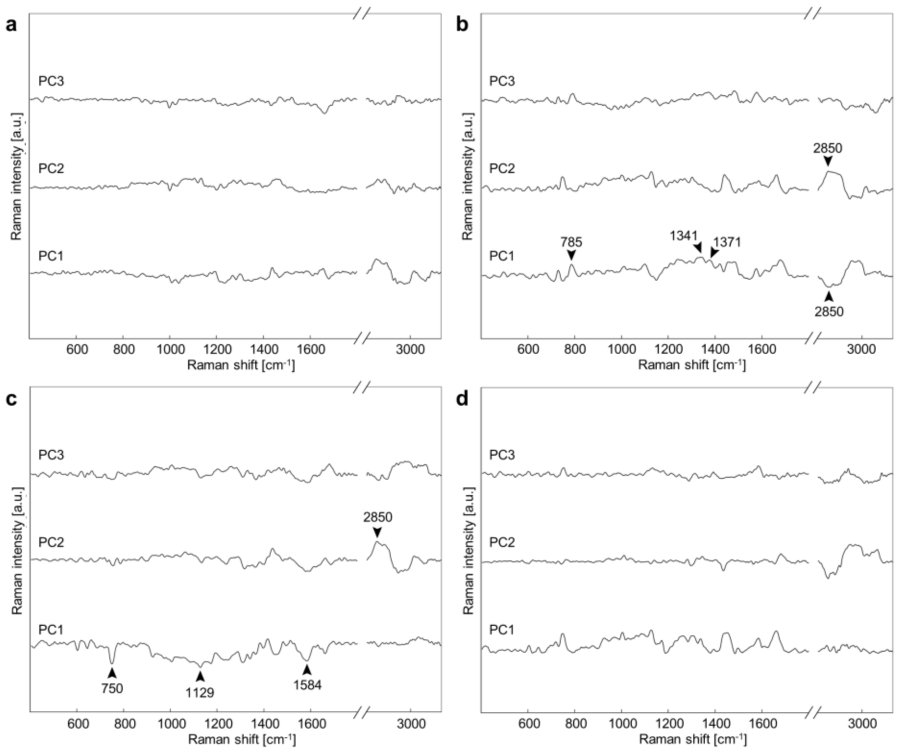

Supplementary Figure 6. Loadings of PCA of Raman spectra grouped by biological entity. Loadings of PCA of **(a)** Aβ plaques, **(b)** cell nuclei, **(c)** cytochrome c, and **(d)** lipids, $n = 3$, $N = 3$. Notable peaks are marked by black arrow tips.

Supplementary Figure 7

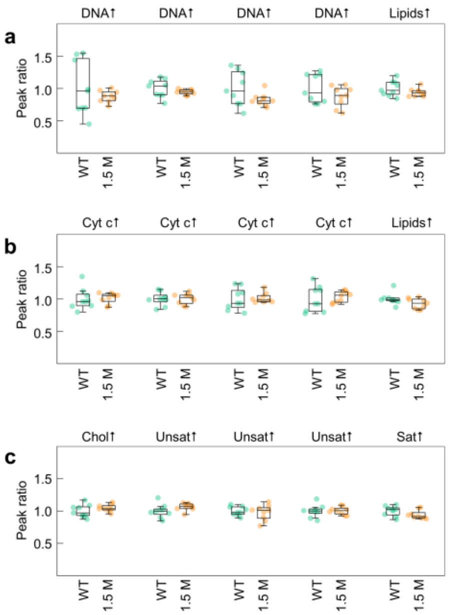

Supplementary Figure 7. Peak ratio analysis for Raman spectra acquired from 1.5 M APP/PS1 mice. Peak ratios for **(a)** cell nuclei spectra, **(b)** cytochrome c spectra, and **(c)** lipid spectra using ratios listed in Supplementary Table 3 are shown, $n = 3$, $N = 3$. Box plot whiskers extend to the most extreme data points which are not considered outliers. Cyt c = cytochrome c, chol = cholesterol, unsat = unsaturation, sat = saturation.

Supplementary Table 1

Supplementary Table 1. Detailed list of Raman peak assignments.

Peaks [cm^{-1}]	Assignment
702	Cholesterol [1,2]
750-752	Cytochrome c [1]
785	DNA, RNA [1]
826	Nitrated tyrosine [3]
1002-1008	Phenylalanine [1,2]
1030-1034	Phenylalanine [1,2]
1070	Fatty acids, lipids [1]
1090	Phosphate [1]
1127	Cytochrome c [4,5]
1224-1242	β-sheet [1,2]
1268	Unsaturated lipids [1]
1310-1315	Cytochrome c [4,6]
1338-1342	DNA, nucleic acids [1]
1369-1375	DNA, nucleic acids [1,7]
1421-1425	DNA, nucleic acids [1,6,8]
1442-1452	Lipids, proteins [1]
1485	DNA, nucleic acids [1]
1584	Cytochrome c [4]
1607-1615	Tyrosine [1,2]
1650 - 1655	Unsaturated lipids and proteins [1]
1665-1670	β-sheet [1,2,9]
1740	Lipid esters [1]
2850	Lipids [1,2]
2883	Lipids [1]
2930-2935	Lipids, Proteins [1]
3010-3015	Unsaturated lipids [1]
3060-3070	Amide [1]

Supplementary Table 2

Supplementary Table 2. Peak ratios for the analysis of Raman spectra by disease state (WT, 1.5 M, 5 M and 10 M APP/PS1).

Peak ratios		Assignment
1668 cm^{-1} / 1444 cm^{-1}	~	β-sheet ↑
785 cm^{-1} / 1003 cm^{-1}	~	DNA ↑
750 cm^{-1} / 1003 cm^{-1}	~	Cytochrome c ↑
2850 cm^{-1} / 2935 cm^{-1}	~	Saturation in lipids ↑

The ratios were calculated using peaks indicative of a biological entity divided by peaks that are not indicative. Arrows indicate that an increasing value of the peak ratio is associated with an increased Raman intensity of the respective biological structure.

Supplementary Table 3

Supplementary Table 3. Peak ratios for the analysis of Raman spectra by biological entity.

Peak ratios		Assignment
**Aβ plaque**		
1667 cm^{-1} / 2935 cm^{-1}	~	β-sheet ↑
3060 cm^{-1} / 2935 cm^{-1}	~	Amide ↑
1003 cm^{-1} / 2935 cm^{-1}	~	Phenylalanine (Phe) ↑
826 cm^{-1} / 2935 cm^{-1}	~	Nitration ↑
1090 cm^{-1} / 2935 cm^{-1}	~	Phosphate ↑
**Cell nuclei**		
785 cm^{-1} / 1003 cm^{-1}	~	DNA ↑
1338 cm^{-1} / 1003 cm^{-1}	~	DNA ↑
1369 cm^{-1} / 1003 cm^{-1}	~	DNA ↑
1424 cm^{-1} / 1003 cm^{-1}	~	DNA ↑
2850 cm^{-1} / 2935 cm^{-1}	~	Lipids ↑
**Cytochrome c**		
750 cm^{-1} / 1003 cm^{-1}	~	Cytochrome (Cyt) c ↑
1129 cm^{-1} / 1003 cm^{-1}	~	Cytochrome (Cyt) c ↑
1310 cm^{-1} / 1003 cm^{-1}	~	Cytochrome (Cyt) c ↑
1584 cm^{-1} / 1003 cm^{-1}	~	Cytochrome (Cyt) c ↑
2850 cm^{-1} / 2935 cm^{-1}	~	Lipids ↑
**Lipids**		
702 cm^{-1} / 720 cm^{-1}	~	Cholesterol (Chol) ↑
1268 cm^{-1} / 1444 cm^{-1}	~	Unsaturation (Unsat) ↑
1650 cm^{-1} / 1444 cm^{-1}	~	Unsaturation (Unsat) ↑
3015 cm^{-1} / 2850 cm^{-1}	~	Unsaturation (Unsat) ↑
2850 cm^{-1} / 2935 cm^{-1}	~	Saturation (Sat) ↑

The ratios were calculated using peaks indicative of different structures of the respective biological entity, divided by peaks that are not indicative, meaning constant, or inversely correlated. Arrows indicate that an increasing value of the peak ratio is associated with an increased Raman intensity of the respective biological structure.

Supplementary References

1. Movasaghi, Z., Rehman, S. & Rehman, I. U. Raman Spectroscopy of Biological Tissues. *Appl. Spectrosc. Rev.* **42**, 493–541 (2007).
2. Niederhafner, P. et al. Monitoring peptide tyrosine nitration by spectroscopic methods. *Amino Acids* **53**, 517–532 (2021).
3. Fonseca, E. A. et al. Micro-Raman spectroscopy of lipid halo and dense-core amyloid plaques: aging process characterization in the Alzheimer's disease APPswePS1ΔE9 mouse model. *Analyst* **146**, 6014–6025 (2021).
4. Russo, V. et al. Key Role of Cytochrome C for Apoptosis Detection Using Raman Microimaging in an Animal Model of Brain Ischemia with Insulin Treatment. *Appl. Spectrosc*. **73**, 1208–1217 (2019).
5. Abramczyk, H., Surmacki, J. M., Brozek-Pluska, B. & Kopec, M. Revision of Commonly Accepted Warburg Mechanism of Cancer Development: Redox-Sensitive Mitochondrial Cytochromes in Breast and Brain Cancers by Raman Imaging. *Cancers* **13**, 2599 (2021).
6. Kunapareddy, N., Freyer, J. P. & Mourant, J. R. Raman spectroscopic characterization of necrotic cell death. *JBO* **13**, 54002 (2008).
7. Ichimura, T. et al. Visualizing the appearance and disappearance of the attractor of differentiation using Raman spectral imaging. *Sci. Rep.* **5**, 11358 (2015).
8. Maiti, N. C., Apetri, M. M., Zagorski, M. G., Carey, P. R. & Anderson, V. E. Raman spectroscopic characterization of secondary structure in natively unfolded proteins: alpha-synuclein. *JACS* **126**, 2399–2408 (2004).
9. A.L.M. Batista de Carvalho, M. Pilling, P. Gardner, J. Doherty & MPM Marques. Chemotherapeutic Response to Cisplatin-like Drugs in Human Breast Cancer Cells Probed by Vibrational Microspectroscopy. In Advanced vibrational spectroscopy for biomedical applications. *Faraday Discuss.* **187**, 273–298 (2016).
