# Supplementary material for: Mapping cellular stress and lipid dysregulation in Alzheimer-related progressive neurodegeneration using label-free Raman microscopy
Source: Commun Biol. 2024 Nov 15;7:1514. doi: 10.1038/s42003-024-07182-6 (PMC11568221; doi:10.1038/s42003-024-07182-6)
Supplement: Supplementary file 2 — Description of Additional Supplementary Files [file 42003_2024_7182_MOESM2_ESM.pdf]

Description of Additional Supplementary Files

File name: Supplementary Data
Description: Data used to plot the graphs in the main text and Supplementary Information
